# Assessing inflammation and predicting relapse in ulcerative colitis is more accurate when performed by IBD specialized gastroenterologists

**DOI:** 10.1093/crocol/otag112

**Published:** 2026-10-08

**Authors:** Madeline Alizadeh, Lauren A George, Ameer Abutaleb, Wenqing Cao, Bruce D Greenwald, Harris T Feldman, Shien Hu, David Hudesman, Frances Onyimba, Suparna Sarkar, Kristen Stashek, Katherine Sun, Uni Wong, Samantha Zullow, Jordan Axelrad, Raymond K Cross

**Affiliations:** Institute for Genome Sciences, University of Maryland School of Medicine, Baltimore, MD, United States; Division of Gastroenterology and Hepatology, Department of Medicine, University of Maryland School of Medicine, Baltimore, MD, United States; Division of Gastroenterology and Hepatology, Department of Medicine, MedStar Washington Hospital Center, Washington, DC, United States; Department of Medicine, George Washington School of Medicine and Health Sciences, Washington, DC, United States; Department of Pathology, NYU Grossman School of Medicine, NYC, NY, United States; Division of Gastroenterology and Hepatology, Department of Medicine, University of Maryland School of Medicine, Baltimore, MD, United States; Department of Medicine, Beth Israel Deaconess Medical Center, Boston, MA, United States; Frederick Gastroenterology Associates, Frederick, MD, United States; Division of Gastroenterology, Department of Medicine, NYU Grossman School of Medicine, NYC, NY, United States; WellSpan Digestive Health, WellSpan York Hospital, York, PA, United States; Department of Pathology, NYU Grossman School of Medicine, NYC, NY, United States; Department of Pathology, University of Pennsylvania, Philadelphia, PA, United States; Department of Pathology, NYU Grossman School of Medicine, NYC, NY, United States; Department of Veterans Affairs, Washington DC Veterans Health Administration, Washington DC, United States; Department of Medicine, Beth Israel Deaconess Medical Center, Boston, MA, United States; Division of Gastroenterology, Department of Medicine, NYU Grossman School of Medicine, NYC, NY, United States; Melissa L. Posner Institute for Digestive Health & Liver Disease at Mercy Medical Center, Baltimore, MD, United States

**Keywords:** ulcerative colitis, histopathology, IBD disease activity, inflammatory bowel disease

## Abstract

**Background:**

Ulcerative colitis is monitored through clinical, laboratory, endoscopic, and histologic measures. Persistence of histologic inflammation despite clinical and endoscopic remission is associated with disease flare.

**Methods:**

We compared the identification of clinically significant histologic inflammation by general and IBD-specialized gastroenterologists (GI) using a simplified binary scoring system and looked at associations with disease relapse.

**Results:**

Both general and IBD-specialized GIs identified significant inflammation associated with relapse, though IBD-specialized GIs scoring had stronger correlations with relapse.

**Conclusions:**

Our findings suggest simple histologic assessment can inform risk stratification, and further work is needed to optimize monitoring patients with histologic inflammation despite endoscopic remission.

## Introduction

Ulcerative colitis (UC), a subtype of inflammatory bowel disease (IBD), is an immune-mediated inflammatory disease of the colon that affects at least 1% of the United States population.^1^ Patients with UC are diagnosed and monitored using a combination of clinical symptoms, laboratories, imaging, endoscopy, and histopathology from mucosal biopsies.^2^ UC-associated histologic inflammation includes lamina propria expansion by a chronic inflammatory infiltrate composed predominantly of plasma cells and eosinophils. Neutrophils are present in active disease, forming crypt abscesses and cryptitis, and when severe, can lead to erosion and ulceration.^3^ Active histologic inflammation may continue despite clinically or endoscopically quiescent disease, and data increasingly suggests histologic activity alone may predict future flares.^4^ Determining clinically significant inflammation on histology is key to identifying patients at highest risk. However, community and referral center pathologists rarely provide validated histologic scoring of histologic activity on biopsy specimens. It has not been determined if a provider’s qualitative assessment of a pathology report is sufficient to predict a future relapse of disease, which could inform a clinical intervention. Given the importance of recognizing histologic activity for treatment planning and monitoring in the real world, we designed this study with the aim of comparing the identification of clinically significant inflammation between non-IBD gastroenterologists (GI) and IBD-specialized GIs, and to assess the association with disease relapse.

## Methods

Patients were identified for inclusion via the protocol described in a prior publication^4^; briefly, a query of patients with UC that had a colonoscopy performed at our two institutions was performed, and records were then manually reviewed to identify patients with a Mayo Endoscopic score of 0 or 1 with available biopsies and accessible pathology reports. To assess the ability to analyze pathology reports and determine whether inflammation is significant, we utilized a simplified scoring method based on the presence of inflammation in pathology reports. Two non-IBD and two IBD attending Gis each at two large IBD centers with at least two years of experience out of fellowship graded inflammation from de-identified pathology reports, scoring each report as 0, representing minimal to no inflammation, or 1, representing the presence of significant inflammation. Pathology reports were de-identified from disease specific information and then sorted into an Excel document and sent to each reader individually, and each independently scored every report from 0 to 1 based on the pathology report. For cases where a discrepancy between graders of the same type occurred, an additional non-IBD or IBD GI served as an adjudicator. In order to assess for intra-observer agreement, 10% of all data from study participants were duplicated randomly and placed back into the data pool. Clinical relapse was defined as experiencing at least one of the following within one year of the endoscopy: a change in medical therapy, corticosteroid prescription, hospitalization, or colectomy. Quantitative and categorical variables were compared using Welch’s *t*-tests and Fisher’s exact test, respectively; comparator groups were those with and without significant inflammation on histology based on both general and IBD-specialized GI assessment. Multiple logistic regression models were developed using Generalized Linear Modeling (GLM) with binomial distribution to assess the relationship between clinical relapse and identification of significant inflammation on histology. Comparisons for disease duration and extent of disease were made with unknown values (NAs) missing, with *P*-values based on comparing known values. For the linear regression, NA’s were removed from the model. All statistics were performed using the R “stats” package in R version 4.3.1.

## Results

Participants were an average of 40.1 years old, 50.6% female, 77.4% White, and 8.2% Black. Duration of disease was an average of 10 years, and most participants were nonsmokers (69.5%), with few current smokers (4.7%) in the cohort (Table 1). Pancolitis was the most commonly observed disease extent (44.4%), with a substantial minority having proctitis (18.6%). Left-sided disease was slightly less common than proctitis among those considered to have significant inflammation (*P* = .006 stratified by general GI, *P* = .002 when stratified by IBD-specialized GI assessment). Stratification by IBD-specialized GI assessment also revealed that participants classified as having significant inflammation were younger (*P* = .004) with shorter UC duration (*P* = 3.87e-04) than those without. There was no difference in clinical characteristics between those who relapsed and those who did not, though UC disease duration trended toward being shorter in those who experience relapse (median 5 vs 8 years, *P* = .052).

**Table 1:** Demographic and clinical characteristics of the patients with ulcerative colitis sub-stratified by inflammation severity based on general vs IBD-specialized gastroenterologist scoring. All bolded p-values are considered statistically significant.

Variable	Total cohort (*n* = 403)	Non-inflamed based on general GI (*n* = 296)	Inflamed based on general GI (*n* = 107)	*P* value	Non-inflamed based on IBD-specialized GI (*n* = 290)	Inflamed based on IBD-specialized GI (*n* = 113)	*P* value
**Age (years; median, IQR, range)**	37.0	37.0	37.0	.373	38.0	33.0	**.004**
	(28, 50)	(28, 50)	(28, 50)		(29, 51.8)	(26, 44)	
	(17, 85)	(17, 85)	(19, 80)		(17, 85)	(17, 80)	
**Sex (F)**	204 (50.6%)	153 (51.7%)	51 (47.7%)	.500	145 (50.0%)	59 (52.2%)	.740
**Race**				.106			.116
** White**	312 (77.4%)	234 (79.1%)	78 (72.9%)		231 (79.7%)	81 (71.7%)	
** Black**	33 (8.2%)	19 (6.4%)	14 (13.1%)		19 (6.6%)	14 (12.4%)	
** Other**	58 (14.4%)	43 (14.5%)	15 (14.0%)		40 (13.8%)	18 (15.9%)	
**Extent**				.006			.002
** E1 proctitis**	75 (18.6%)	50 (16.9%)	25 (23.4%)		46 (15.9%)	29 (25.7%)	
** E2 left-sided colitis**	142 (35.2%)	108 (36.5%)	34 (31.8%)		106 (36.7%)	36 (31.9%)	
** E3 pancolitis**	179 (44.4%)	131 (44.3%)	48 (44.9%)		131 (45.2%)	48 (42.5%)	
** Unknown**	7 unknown (1.7%)	7 unknown (2.4%)			7 unknown (2.4%)		
**UC Duration (years; median, IQR, range)**	8	8	6	.139	8	5	**3.873e-04**
	(2, 15)	(2, 15)	(2, 15)		(3, 16)	(1.3, 11.8)	
	(0, 52)	(0, 52)	(0, 41)		(0, 52)	(0, 41)	
	25 unknown	17 unknown	8 unknown		22 unknown	3 unknown	
**Smoking status**				.771			.124
** Never**	280 (69.5%)	205 (69.3%)	75 (70.1%)		193 (66.6%)	87 (77.0%)	
** Former**	104 (25.8%)	78 (26.4%)	26 (24.3%)		82 (28.3%)	22 (19.5%)	
** Current**	19 (4.7%)	13 (4.4%)	6 (5.6%)		15 (5.2%)	4 (3.5%)	
**Relapse (defined as one of the four criteria)**	98 (24.3%)	61 (20.6%)	37 (34.6%)	**.006**	53 (18.3%)	45 (39.8%)	**1.524e-05**

At the univariate level, relapse of disease occurred in 24.3% and was more likely in those scored as having significant inflammation, both by general GIs (*P* = .006) and IBD-specialized GIs (*P* ≤ .001; Table 1). The + LR vs − LR for those scored by IBD-specialized GIs was 2.02 and 0.69, compared to 1.72 and 0.79 for general GIs, with general ORs of 2.90 vs 2.18, respectively (Table 2). Multivariate logistic regressions controlling for disease location and UC duration, as well as race, were developed; differences in race between those with vs without significant inflammation on the pathology report approached significance, indicating race should be accounted for in the model. Even when controlling for the aforementioned factors, both general GIs and IBD GIs assessment of significant inflammation were highly associated with relapse, with a slight increase in association with IBD GIs’ assessments (OR = 2.108 and 2.763, *P* = .004 and 7.32e-05, AIC = 424.76 and 417.16, respectively; Table 3).

**Table 2:** Likelihood (LR) and univariate odd’s ratios (DOR) of relapse based on inflammation scoring.

Ratio	General gastroenterologists	IBD-specialized gastroenterologists
**+LR**	1.72	2.02
**−LR**	0.79	0.69
**DOR**	2.18	2.90

**Table 3:** Multivariate models of general vs IBD-specialized gastroenterologists’ inflammation scoring as predictors of relapse. All bolded values are considered statistically significant.

Variable	OR	95% CI	*P* value	Variable	OR	95% CI	*P* value
**General GI model**				**IBD-Specialized GI model**			
**Phenotype E2: E1**	0.869	(0.447, 1.718)	.683	**Phenotype E2: E1**	0.908	(0.463, 1.811)	.782
**Phenotype E3: E1**	1.154	(0.620, 2.199)	.657	**Phenotype E3: E1**	1.222	(0.651, 2.351)	.539
**Race Black: White**	1.119	(0.430, 2.688)	.808	**Race Black: White**	1.049	(0.398, 2.551)	.919
**Race Other: White**	1.607	(0.840, 3.006)	.143	**Race Other: White**	1.606	(0.834, 3.025)	.148
**UC duration**	0.984	(0.958, 1.008)	.218	**UC duration**	0.990	(0.963, 1.013)	.418
**General GI prediction of inflammation**	**2.108**	**(1.264, 3.501)**	**.004**	**IBD-specialized GI prediction of inflammation**	**2.763**	**(1.673, 4.578)**	**7.32e-05**

## Discussion

We found both general and IBD-specialized GIs can qualitatively interpret histologic inflammation severity using a routine histopathology report, without formal histologic scoring, though IBD-specialized GI interpretation is more strongly predictive of future flares. Even with normal endoscopic findings, histologic disease activity in UC is associated with future colorectal neoplasia,^5^ hospitalization, and surgery.^6^ Although histologic disease activity is associated with less invasive markers such as fecal calprotectin,^7^ sensitivity and specificity are not high enough to consider them as an adequate substitute for mucosal sampling. However, appropriate evaluation of histologic pathology is required for clinically actionable interpretation; this is made more difficult by limited reporting of histologic scores, variation in scoring indices, inconsistencies in mucosal sampling procedures, and the lack of agreement in defining histologic remission and activity.^6^ We were limited by the fact that we did not have data on validated scoring indices (eg, Robarts) to compare to in our study population, and external applicability may be limited as there may be less standardized pathology reporting in the community setting. Despite these challenges, we found that GIs at an academic center could identify clinically significant inflammation, regardless of IBD-specialization. Importantly, this assessment predicted future clinical relapse. Future research is needed to validate our findings and develop a standardized, accessible, simple scoring index that can be utilized in clinical practice and to determine what actions are needed when significant histologic inflammation is present (ie, enhanced monitoring or escalation of treatment).

## Data Availability

The data that support the findings of this study are available from the corresponding author upon reasonable
